# Proteomics Analysis for Finding Serum Markers of Ovarian Cancer

**DOI:** 10.1155/2014/179040

**Published:** 2014-08-31

**Authors:** Yushan Cheng, Chongdong Liu, Nawei Zhang, Shengdian Wang, Zhenyu Zhang

**Affiliations:** ^1^Beijing Chaoyang Hospital Affiliated with Capital Medical University, Beijing 100020, China; ^2^Institute of Biophysics, Chinese Academy of Sciences, Beijing 100101, China

## Abstract

A combination of peptide ligand library beads (PLLB) and 1D gel liquid chromatography-mass spectrometry/mass spectrometry (1DGel-LC-MS/MS) was employed to analyze serum samples from patients with ovarian cancer and from healthy controls. Proteomic analysis identified 1200 serum proteins, among which 57 proteins were upregulated and 10 were downregulated in the sera from cancer patients. Retinol binding protein 4 (RBP4) is highly upregulated in the ovarian cancer serum samples. ELISA was employed to measure plasma concentrations of RBP4 in 80 samples from ovarian cancer patients, healthy individuals, myoma patients, and patients with benign ovarian tumor, respectively. The plasma concentrations of RBP4 ranging from 76.91 to 120.08 ng/mL with the mean value 89.13 ± 1.67 ng/mL in ovarian cancer patients are significantly higher than those in healthy individuals (10.85 ± 2.38 ng/mL). Results were further confirmed with immunohistochemistry, demonstrating that RBP4 expression levels in normal ovarian tissue were lower than those in ovarian cancer tissues. Our results suggested that RBP4 is a potential biomarker for diagnostic of screening ovarian cancer.

## 1. Introduction

Ovarian cancer is the most lethal type of gynecological cancer in the world [1]. Most patients (75%) are diagnosed with advanced stage disease (FIGO stage III/IV) with a 5-year overall survival rate of less than 30% [2]. In contrast, patients with FIGO stage I ovarian cancer have a survival rate of approximately 95% [3, 4]. The high frequency and poor prognosis of ovarian cancer emphasizes the need to identify diagnostic markers for ovarian cancer. The most frequently used marker of ovarian cancer is CA125 which has a poor sensitivity of 65% [5]. Extensive studies have been carried out to identify serum/plasma biomarkers for ovarian cancer diagnosis [6, 7]. Petricoin [8] used the surface-enhanced laser desorption/ionization (SELDI)-TOF-MS to classify an independent set of 116 unmasked serum samples: 50 from women with ovarian cancer and 66 from unaffected women or those with nonmalignant disorders. The algorithm identified a cluster pattern that completely segregated cancer from noncancer. Lin et al. [9] studied the serum proteins from 35 women with ovarian cancer and 30 age-matched disease-free controls with SELDI-TOF-MS and identified four specific protein peaks in plasma of women with ovarian cancer, but not in controls, with molecular masses of 6190.48, 5147.06, 11522.6, and 11537.7 Dalton. So far, more than 30 serum markers have been evaluated alone or in combination with CA125, for example, lysophosphatidic acid, osteopontin, ovarian carcinoma associated antigen, and HE4 for testing their qualifications as biomarkers [10, 11]. One recent study reported higher specificity and sensitivity for early detection of ovarian cancer by using a combination of 4 markers (APAO1, a truncated form of transthyretin, a fragment of inter-*α*-trypsin inhibitor heavy chain H4, and CA125) compared to conventional marker CA125 alone [7]. However, due to limitations in SELDI-TOF-MS and other approaches and the complexity of serum/plasma proteome, few serum/plasma proteins have been developed into diagnostic markers of ovarian cancers in the clinical settings.

One challenge to identify these candidate biomarkers is that there is an extensive dynamic concentration range of proteins in the biofluids. For example, the range can reach up to 12 orders of magnitude for plasma/serum proteome. In serum the primary component is serum albumin which is the major carrier and transporter at a concentration of 35~50 g/L, representing 60–72% of the total protein content. But most of the important indicators changing in physiological states may be possibly present at <1 pg/mL, such as cytokines and tissues leakage proteins. In general, 85 percent of the human serum proteins by mass are comprised of six high abundance proteins including albumin, immunoglobulins, transferrin, haptoglobin, and *α*-1-antitrypsin. These components may mask the mass spectra of the interesting low abundance proteins. Methods have been developed to deplete the high abundance proteins derived from serum/plasma, such as immunodepletion, organic precipitation, affinity purification, and solid phase extraction [12, 13]. Peptide ligand library affinity chromatography (PLLB) is a novel method for capturing and identifying the low abundance proteins [14]. In this method, a solid-phase combinatorial library of hexapeptides is coupled, via a shorter spacer, on poly (hydroxymethecrylate) beads, by a modified Merrifield approach. The hexapeptide ligands are synthesized from natural amino acid so the library contains a population of linear hexapeptide amounting up to few dozen millions of different ligands. It means that an appropriate volume of beads should contain a partner able to interact with a very large number of proteins present in a complex proteome. Sennels et al. [15] have reported a large scale proteomic study of human serum using peptide library beads and mass spectrometry. Analysis of the eluates from this combinatorial library resulted in the identification of 1559 proteins including a large number of low abundance proteins.

In the present study, a combination of PLLB and 1 G Gel-LC-MS/MS was used to identify differences in proteins from patients with ovarian cancer as compared to healthy controls. 1200 serum proteins were identified and 67 proteins were found to be differentially expressed between serum samples from healthy controls and ovarian cancer patients.

## 2. Materials and Methods

### 2.1. Subjects and Materials

The study protocol was approved by the Ethics Committee of Beijing Chaoyang Hospital. All patients in this study were of Chinese origin. All the subjects were from the Department of Obstetrics and Gynecology in Chaoyang Hospital affiliated with Capital Medical University in Beijing, China. All the patients involved signed informed consent forms. From the samples available, we selected serum samples from 25 ovarian cancer patients as experimental group, 20 serum samples from women with benign ovarian tumor, 20 patients with myoma, and 25 serum samples from normal, apparently healthy women as control group during March 2007 to July 2010. Patients with diabetes, kidney disease, liver disease, or other cancers were excluded in the present study.

### 2.2. Sample Collection and Preparation

#### 2.2.1. Blood Collection

Blood samples were collected at the time of preliminary diagnosis before any treatments. The diagnosis was histologically confirmed after surgery. Serum was obtained from the peripheral blood by centrifugation at 4000 rpm for 10 minutes at 4°C within 2 hours of the collection and stored at −80°C until analyses.

#### 2.2.2. Tissue Collection

To assess whether or not the expression of RBP4 was altered in association with the presence of ovarian cancer, matching ovarian tissue samples were collected from normal ovary. Control tissues were collected from patients undergoing surgery as a result of benign ovarian tumor. Case tissues were removed at the time of tumor cytoreduction surgery issues and frozen in liquid nitrogen then stored at −80°C. Where possible, blood and tissue collected from the same patient was used for both IHC and ELISA analysis.

### 2.3. Protein Separation, In-Gel Digestion, and LC-MS/MS Analysis

Serum samples from 5 ovarian cancer patients and 5 health controls were pooled together, respectively. Depletion of high abundance proteins were carried out based on the well-established protocol [16]. Briefly, 300 *μ*L of the pooled serum samples was centrifuged to eliminate particles in suspension. 8 mg of the PLLB resin (BioRad Laboratories, Hercules, CA) was suspended in 100 *μ*L of 50% methanol for 10 minutes and was washed three times with PBS solution (pH = 7.4). Then the pooled serum samples were incubated with the PLLB resin at room temperature (22–25°C) on a soft shaker for 2 hours. After removing the unbound fraction, the PLLB resin was washed three times with PBS solution again. Proteins were eluted from the beads by incubating with LDS sample buffer (Invitrogen, Grand Island, NY) at 100°C for 5 minutes.

Proteins were separated on a 4–12% gradient Tris-Glycine SDS-gel (Invitrogen, Grand Island, NY) and were stained with colloidal Coomassie Blue (Invitrogen, Grand Island, NY). Each lane was cut into 15 slices and each gel slice was reduced with 10 mM dithiothreitol (Calbiochem, San Diego, CA) and alkylated with 100 mM iodoacetamide (Sigma, St. Louis, MO). Then in-gel digestion was carried out with the sequence grade modified trypsin (Promega, Fitchburg, WI) in 50 mM ammonium bicarbonate at 37°C overnight. The peptides were extracted twice with 1% trifluoroacetic acid in 50% acetonitrile aqueous solution for 30 minutes.

For LC-MS/MS analysis, each digestion product was separated by a 60 min gradient elution at a flow rate of 0.250 *μ*L/min with the Dionex 3000 nano-HPLC system, which is directly interfaced with the Thermo LTQ-Orbitrap mass spectrometer. The analytical column was a home-made fused silica capillary column (75 *μ*m ID, 150 mm length; Upchurch, Oak Harbor, WA) packed with C-18 resin (300 A, 5 *μ*m, Varian, Lexington, MA). Mobile phase A consisted of 0.1% formic acid, and mobile phase B consisted of 100% acetonitrile and 0.1% formic acid. The LTQ-Orbitrap mass spectrometer was operated in the data-dependent acquisition mode using the Xcalibur 2.0.7 software and there is a single full-scan mass spectrum in the Orbitrap (400–1800* m/z*, 30,000 resolution) followed by 6 data-dependent MS/MS scans in the ion trap at 35% normalized collision energy.

### 2.4. Data Processing and Quantitative Analysis

The MS/MS spectra from each LC-MS/MS run were converted from RAW file format to DTA files using BioWorks 3.3.1 (Thermo-Fisher, San Jose, CA). The DTA files were searched against the human IPI database using an in-house Mascot searching algorithm. The following search parameters were used in all of the Mascot searches: maximum of 1 missed trypsin cleavages, cysteine carbamidomethylation as fixed modification, and methionine oxidation as the variable modification. The maximum error tolerance was 10 ppm for MS and 1.2 Da for MS/MS. Proteins were designated as “hits” only when the Mascot score was more than 30 and there were at least 2 unique peptides matches. When several proteins matched the same sets of peptides, only the protein with the greater percentage of coverage was selected. Quantitation of protein expressions by spectral counts for each identified proteins was carried out using an in-house developed Perl script. Significance was regarded only when the ratio of spectral counts between two groups were more than 2 or less than 0.5. Extracted ion currents for selected peptides were also used to quantify the protein concentrations from different samples. Confirmation of some of the differentially expressed proteins in the present study was also carried out with isotope-encoded peptides corresponding to the tryptic peptides of the selected proteins. The same amount of pooled serum samples from ovarian cancer and healthy controls were treated with PLLB resin, respectively, using the same protocol described above. The eluted proteins were separated on 1D-SDS-PAGE followed by in gel digestion. Then the tryptic peptides from each gel band were pooled and spiked with the isotope-encoded synthetic peptides as the internal standards followed by LC-MS/MS analysis with LTQ-Orbitrap mass spectrometer.

### 2.5. ELISA Assay Analysis

The study included 20 ovarian cancer patients; the control groups were comprised of healthy controls (*n* = 20), myoma group (*n* = 20), and benign ovarian tumor group (*n* = 20). RBP4 concentrations in serum samples from four groups were measured using an adapted protocol from a commercially available ELISA kit by R&D Systems (Minneapolis, MN, USA). For RBP4, rabbit polyclonal anti-human RBP4 (Santa Cruz Biotechnology, CA, USA) capture antibody was immobilized in a 96-well clear polystyrene plate by incubating 50 *μ*L of 2.0 ng/*μ*L capture antibody in PBS (NaCl 137 mmol/L, KCl 2.7 mmol/L, Na_2_HPO_4_ 4.3 mmol/L, KH_2_PO_4_ 1.4 mmol/L, and pH 7.4) overnight. The plates were washed three times with washing buffer (5 mmol/L Tris, 150 mmol/L NaCl, 0.05% Tween20, pH 7.8), after which the plate was blocked by adding 50 *μ*L of Reagent Diluent (1% BSA in PBS) to each well and incubated with shaking at room temperature for 60 min. The plates were then washed three times with washing buffer and incubated with 50 *μ*L per well of RBP4 standards or serum samples with shaking at room temperature for 2 h. RBP4 standards and serum samples were diluted in Reagent Diluent with all serum samples diluted 5-fold. After incubation, the plates were washed six times with washing buffer and incubated with 90 *μ*L per well of biotinylated rabbit anti-human RBP4 detection antibody solution (100 pg/*μ*L detection antibody in Reagent Diluent) with shaking at room temperature for 2 h. After washing the plates six times with washing buffer, 50 *μ*L of streptavidin-conjugated horseradish peroxidase solution (diluted 200-fold in Reagent Diluent) was added to each well and incubated for 30 min with shaking at room temperature. A final wash of six times with washing buffer was followed by the addition of 100 *μ*L Tetramethylbenzidine (Zhongshan Goldenbridge Biotechnology, Beijing, China) per well and incubated with shaking at room temperature for 30 min. The chromogenic reaction was stopped with the addition of 50 *μ*L of 2 mol/L hydrochloric acid solution per well. Subsequently, the absorbance of each well was measured with the Wallac Envision 2103 Multilabel Reader (PerkinElmer) at 450 nm. Final serum concentrations were calculated by multiplying with the dilution factor. All samples were analyzed in triplicate.

### 2.6. Immunohistochemistry

The expression of RBP4 in ovarian tissues was assessed using PV-9000(standard polymer detection system) for immunohistological staining. Tissue samples were fixed in sodium phosphate buffer containing 10% formalin. Frozen tissue sections 5 *μ*m thick were cut at −23°C using a cryostat. Tissue sections were fixed in acetone for 15 min at −20°C then washed in TBS. After 1-2 d of fixation, selected tissue blocks were processed and embedded in paraffin. The sections were deparaffinized, rehydrated, and incubated with 3%H_2_O_2_ in methanol for 30 min to quench endogenous peroxidase activity. After a short rinse, the sections were boiled in water bath for 15–20 min in citrate buffer. Following cooling and rinsing, 50 *μ*L rabbit polyclonal RBP4 antibody (Santa Cruz Biotechnology, CA, USA) was applied on the sections for 30 min and incubated overnight at 4°C then shaking at room temperature for 30 min. Antibody binding was amplified using biotin and streptavidin HRP for 10 minutes each and the complex was visualised using DAB. PBS was substituted for the antibody as a negative control. The negative controls for IHC were carried out under the same experimental conditions. ALL sections were assessed microscopically for positive DAB staining. The immunostained sections were examined using Leica DMLA light microscope (Leica Microsystems, Wetzlar, Germany) to assess the prevalence of positive cases and the localization of immunostaining within the tissues. Tumor cells with unequivocal staining of the granular cytoplasm were considered positive. RBP4 expression was evaluated by computer assessment method. The expression level of RBP4 was scored on the basis of the intensity of staining of the cytoplasm of the cells over the entire section.

### 2.7. Statistical Analysis

Statistical analysis was performed using SPSS software, version 13.0 (SPSS Inc., Chicago, IL). Data of ELISA were presented as *x* ± *s*. For all statistical comparisons, *P* < 0.05 was taken as statistically significant.

## 3. Results

### 3.1. Identification of Proteins by 1D Gel LC-MS/MS with PLLB

**Figure 1:**
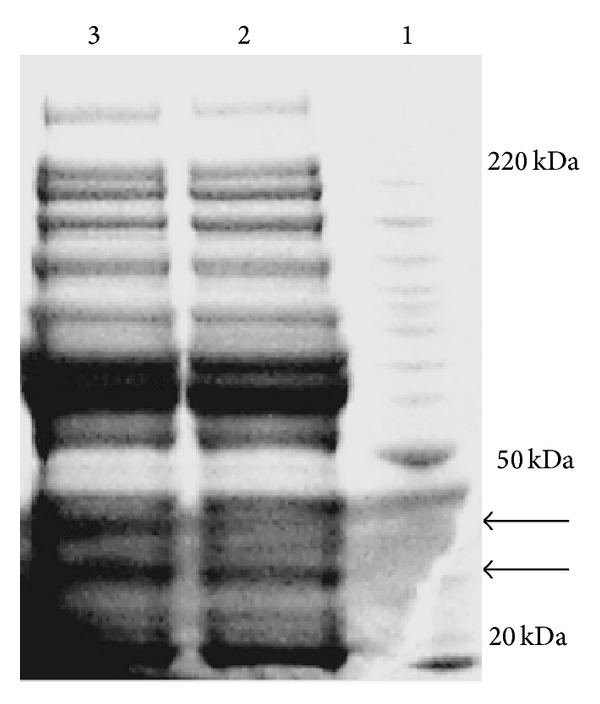
1D-SDS-P1D-SDS-PAGE gel images of serum proteins after PLLB treatment. Lane 1: molecular weight marker. Lanes 2 and 3: serum proteins of healthy and ovarian cancer eluted from the PLLB.

The serum samples from cancer patients and healthy women were treated with PLLB, and the elutes were separated on 1D-SDS-PAGE (Figure 1). In comparison to the untreated serum samples, abundance serum proteins such as albumin and IgG were greatly reduced by PLLB treatment. Each lane was cut into 15 pieces, in gel digested, and analyzed by LC-MS/MS. From each lane, we identified approximately 1200 unique proteins in ovarian cancer as well as in healthy controls. The false positive rate for proteins identified with Mascot, as calculated by the decoy database search, was estimated to be 2%. Although the numbers of proteins identified are not as high as what have been reported, our results are more reliable since the MS measurement was carried out with LTQ-Orbitrap mass spectrometer with the mass measurement error less than 10 ppm. The studies carried out with LTQ usually use a 3 Da as mass measurement error, which greatly increases the false positive rate.

### 3.2. Differentially Expressed Serum Proteins

When comparing Lane 2 from healthy women to Lane 3 from ovarian cancer patients, staining intensities show a few differences in several gel bands, as shown in Figure 1. Spectral count was used to quantify the expression levels of proteins in the selected gel bands as well as the whole lane. Spectral count uses the number of spectra that have been assigned to a specific protein, to quantify the relative abundance of a protein from two samples, and it has been widely applied to biological systems. For proteins with low spectra counts, extracted ion currents were also used for quantitation. It has been noticed in our previous studies that ratios of protein expressions are rather semiquantitative based on spectra counts or extracted ion current. Using 2-fold or more changes as a determinant, we have identified that 57 proteins were upregulated and 10 proteins were downregulated in ovarian cancer patients as compared with those in healthy women (Table 1).

### 3.3. ELISA Analysis of RBP4

**Figure 2:**
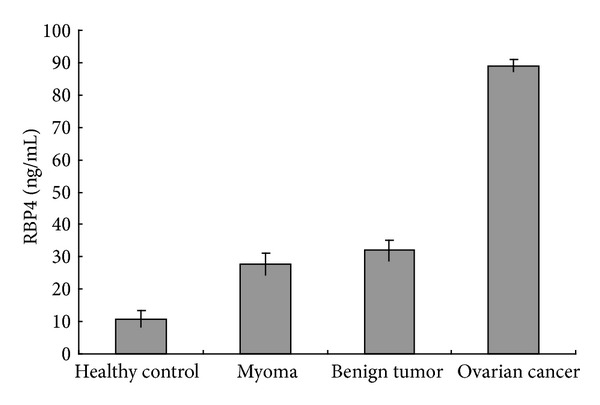
Mean levels of RBP4 serum content measured with ELISA, showing that the expression of RBP4 in patients with ovarian cancer was significantly upregulated compared with the controls (*P* < 0.01).

Baseline characteristics of the 80 patients are listed in Table 2. Measurements of median BMI and age were similar matched between the four groups to avoid affecting the outcome. The distributions of RBP4 in the four serum cohorts studied are shown in Figure 2. As shown in Table 3 RBP4 was expressed in the four groups. Comparisons between the four groups were further detailed in Table 4. Compared with the other three groups, RBP4 levels as detected by ELISA were significantly higher (3–8 times higher) in cancer patient sera (*P* < 0.05). We found upregulation (about 3 times higher) of RBP4 in sera of benign ovarian tumor patients and myoma patients from healthy control patients (*P* < 0.05), while no significant difference was detected between RBP4 in the sera from patients with ovarian tumor and myoma (*P* > 0.05).

### 3.4. Validation by Immunohistochemistry

**Figure 3:**
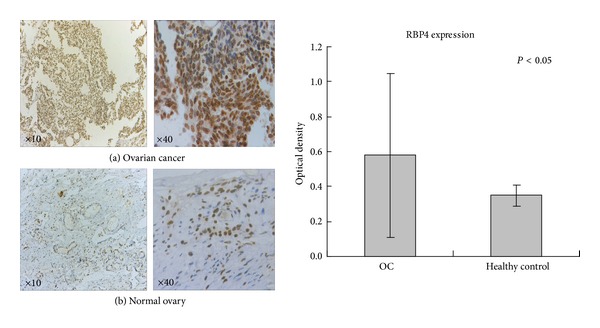
Immunohistochemistry for RBP4. Tissues from ovarian cancer (a) and normal ovaries (b) were stained for RBP4. Original magnification ×10 (low power) or ×40 (high power).

Immunohistochemistry analysis was carried out on ovarian cancer tissues and normal ovarian tissues (Figure 3). The positive expression of RBP4 in cancer tissues shown in brown is significantly increased as compared to the normal ovarian tissue, which shows weak IHC staining (Figure 3(b)). Further analysis indicates that optical density of RBP4 positive expression is 0.58 ± 0.47 for cancer tissues and 0.35 ± 0.06 for normal ovarian tissues.

## 4. Discussion 

Ovarian cancer is one of the most deadly cancers with a 5-year overall survival rate less than 30%. New biomarkers are urgently needed to improve diagnosis of ovarian cancer and to increase the survival rates of patients. Over the last decade, proteomics has been widely applied to biomarker discovery [17, 18]. Rai et al. selected a seven-marker model to discriminate between ovarian cancer and healthy patients [19], including transferrin, haptoglobin, and immunoglobulin heavy chain. Ahmed et al. identified isoforms of haptoglobin-1 precursor (HAP1) and correlated this with immunohistochemistry in tissue samples [20]. Ye et al. identified haptoglobin alpha subunit to be upregulated in ovarian cancer patients [21].

RBP4 is an adipokine secreted by adipose tissue and liver and contributes to insulin resistance (IR) [22]. Remarkably, elevated RBP4 levels were directly correlated with body mass index (BMI), insulin resistance, and impaired glucose homeostasis and were inversely correlated with glucose transporter-4 levels in adipocytes [23]. Moreover, RBP4 stimulates hepatic gluconeogenesis and inhibits insulin signaling in the muscle [24]. These studies establish that RBP4 plays an important role in diabetes, cardiovascular diseases, kidney diseases, and metabolic syndrome [25]. Preliminary investigation showed that men and women with a BMI of 40.0 and above had a death rate from all cancers combined of 52%, which was 88% higher than their normal-weight counterparts, indicating that RBP4 also plays a role in cancer. Indeed, a recent study showed a highly significant increase of RBP4 level in the pancreatic cancer [26]. Studies also showed that RBP4 were overexpressed in the head and neck squamous cell carcinoma, breast cancer, and colon adenocarcinoma tissues [27]. On the other hand, RBP4 exerts its main functions with ROH and TTR as a trimer through transported specific rake tissues and organs in the body [28, 29]. Existing research shows that TTR can be used as a member of the ovarian tumor marker spectrum [7, 30].

In the present study, we applied the PLLB to deplete abundance proteins in serum and use label free quantitation to find differentially expressed serum proteins between ovarian cancer patients and healthy individuals. We identified about 1200 serum proteins, among which 67 proteins are differentially expressed (Table 2). Several of these proteins are already reported in the literature as related to ovarian cancer. For example, the study by Xu and colleagues demonstrates that LPA levels in the plasma were elevated in women with ovarian cancer [31]. We carried out western blot analysis (data not shown) and identified that Retinol-binding protein 4 (RBP4) was upregulated in sera from cancer patients. Proteomic analysis and ELISA measurement of the serum RBP4 levels showed that RBP4 levels were significant higher in ovarian cancer patients than those in healthy individuals, and RBP4 levels in patients with benign ovarian tumor and myoma were lower than those in cancer patients, but higher than those in the healthy individuals. However, Lorkova et al. identified that retinol-binding protein 4 was decreased in sera of epithelial ovarian cancer patients [32]. Several factors are attributed to differences observed from different proteomic analysis. For example, experimental bias was present, in which control samples and cancer samples were not randomized when they were analyzed on the MS; changes in MS stabilities over time caused statistically significant differences in samples; and different sample preparation conditions [33, 34]. In our study, the results were confirmed by western blotting and immunohistochemistry. We are in the process to carry out a clinical analysis with a large sample size of 100 patient samples that will allow us to further validate our results. Knowing that states of chronic low grade inflammation are associated with increased RBP4 levels [35], so the explanation could be that altered adipokine together with activation of the inflammatory system could promote the development and progression of cancer independently on insulin resistance [36].

CA125 has been the useful marker for ovarian cancer early diagnosis, but it was reported that CA125 was also elevated in other benign and malignant diseases [37, 38]. Given the current progress regarding combinatory multiple ovarian cancer markers [10, 39], we are investigating the potential to use RBP4 as an adjunct markers in combination with CA125 for ovarian cancer diagnosis.

## Figures and Tables

**Table 1:** Characteristics of all the study subjects BMI stands for body mass index (BMI).

	Healthy control (*n* = 20)	Myoma (*n* = 20)	Benign tumor (*n* = 20)	Ovarian cancer (*n* = 20)
Median age (range, y)	41 (31–59)	45 (26–57)	43 (20–48)	53 (40–62)
Median BMI	23.8	24.2	23.1	24.6

**Table 2:** Serum proteins differentially expressed between patients with ovarian cancer and healthy individuals.

Protein ID	Protein name	OC/HC	Mass
Upregulated			
IPI00657670	AHSG alpha-2-HS-glycoprotein precursor	2.0	40098
IPI00022434	ALB isoform 1 of serum albumin precursor	1.1	71317
IPI00021841	APOA1 apolipoprotein A-I precursor	1.6	30759
IPI00021854	APOA2 apolipoprotein A-II precursor	2.4	11282
IPI00847179	APOA4 apolipoprotein A-IV precursor	2.4	45344
IPI00022229	APOB apolipoprotein B-100 precursor	2.2	516666
IPI00021855	APOC1 apolipoprotein C-I precursor	2.0	9326
IPI00021857	APOC3 apolipoprotein C-III precursor	1.8	10846
IPI00006662	APOD apolipoprotein D precursor	1.9	21547
IPI00021842	APOE apolipoprotein E precursor	2.1	36246
IPI00186903	APOL1 isoform 2 of apolipoprotein-L1 precursor	2.0	45957
IPI00030739	APOM apolipoprotein M	4.5	21582
IPI00006608	APP isoform APP770 of amyloid beta A4 protein precursor	2.0	87914
IPI00027507	ARFIP1 isoform B of arfaptin-1	3.0	41770
IPI00218982	BRCA1 breast cancer type 1 susceptibility protein	5.8	210101
IPI00075013	C1QTNF1 complement C1q tumor necrosis factor-related protein 1 precursor	2.2	32065
IPI00186808	CFHR5 complement factor H-related 5	2.4	69411
IPI00552578	CFP properdin precursor	3.0	53751
IPI00400826	CLU clusterin isoform 1	1.1	58537
IPI00013212	CSK tyrosine-protein kinase CSK	2.3	51242
IPI00032311	DADB-112B14.11 complement component 4B	1.5	194167
IPI00328249	EIF2AK1 isoform 1 of eukaryotic translation initiation factor 2-alpha kinase 1	1.9	71632
IPI00296534	FBLN1 isoform D of fibulin-1 precursor	1.6	81315
IPI00293925	FCN3 isoform 1 of ficolin-3 precursor	3.7	33395
IPI00021891	FGG isoform gamma-B of fibrinogen gamma chain precursor	2.5	52106
IPI00298497	FN1 isoform 1 of fibronectin precursor	1.2	266034
IPI00555812	GC vitamin D-binding protein precursor	4.5	54526
IPI00025426	GPX3 glutathione peroxidase 3 precursor	1.8	25774
IPI00012391	HABP2 hyaluronan-binding protein 2 precursor	2.8	64740
IPI00299435	HBA1; HBA2 hemoglobin subunit alpha	3.0	45371
IPI00218660	HSPG2 basement membrane-specific heparan sulfate proteoglycan core protein precursor	1.0	479248
IPI00021885	IGKV1-5 IGKV1-5 protein	1.2	26034
IPI00383338	ITIH1 inter-alpha-trypsin inhibitor heavy chain H1 precursor	1.1	10178
IPI00000075	LGALS3BP galectin-3-binding protein precursor	2.9	66202
IPI00029168	LPA lysophosphatidic acid	2.0	514737
IPI00220249	LTBP1 latent-transforming growth factor beta-binding protein	2.0	180984
IPI00294842	MASP Mannan-binding lectin serine protease	2.0	21129
IPI00743335	MYO1C myosin IC isoform a	2.0	122461
IPI00022446	PF4 platelet factor 4 precursor	5.0	11123
IPI00019580	PLG plasminogen precursor	3.1	93247
IPI00025190	PON1 serum paraoxonase/arylesterase 1	1.1	39895
IPI00022445	PRG4 isoform A of proteoglycan-4 precursor	4.0	152238
IPI00015614	PRSS3 isoform A of trypsin-3 precursor	2.0	33276
**IPI00022420**	**RBP4 plasma retinol-binding protein precursor**	**3.0 **	**23337**
IPI00019399	SAA4 serum amyloid A-4 protein precursor	4.5	14854
IPI00629921	SERPING1 plasma protease C1 inhibitor precursor	3.1	55347
IPI00019176	SPP2 secreted phosphoprotein 24 precursor	3.2	24607
IPI00020194	TAF15 isoform Short of TATA-binding protein-associated factor	2.2	61749
IPI00296099	THBS1 thrombospondin-1 precursor	1.7	133291
IPI00018769	THBS2 thrombospondin-2 precursor	1.8	133749
IPI00022432	TTR transthyretin precursor	3.7	15991
IPI00298971	VTN vitronectin precursor	1.8	55069

Downregulated			
IPI00021812	AHNAK neuroblast differentiation-associated protein AHNAK	0.2	629213
IPI00020567	ARHGAP1 Rho GTPase-activating protein 1	0.1	50461
IPI00294834	ASPH aspartyl/asparaginyl beta-hydroxylase	0.3	86266
IPI00479116	CPN2 carboxypeptidase N subunit 2 precursor	0.5	61431
IPI00074148	DST dystonin isoform 1	0.1	632532
IPI00003351	ECM1 extracellular matrix protein 1 precursor	0.4	62232
IPI00178352	FLNC isoform 1 of filamin-C	0.6	293344
IP00022479	HERC1 probable E3 ubiquitin-protein ligase HERC1	0.1	538918
IPI00550090	JMJD3 jumonji domain containing 3, histone lysine demethylase	0.4	182243
IPI00398728	RP1L1 isoform 1 of retinitis pigmentosa 1-like 1 protein	0.1	263287
IPI0006146	SAA2 serum amyloid A2 isoform a	0.3	11277
IPI00027191	SAA3P putative serum amyloid A-3 protein	0.1	13489
IPI00553177	SERPINA1 isoform 1 of alpha-1-antitrypsin precursor	0.7	46878
IPI00470627	SHPRH SNF2 histone linker PHD RING helicase isoform a	0.5	195906

**Table 3:** Concentrations of RBP4 in ovarian cancer patients, patients with benign ovarian tumors and myoma, and healthy individuals.

Group	Number	Mean ± SD (ng/mL)
Healthy control	20	10.85 ± 2.83
Myoma	20	27.70 ± 3.40
Benign tumor	20	31.97 ± 3.15
Ovarian cancer	20	89.13 ± 1.68

Data are described as mean ± standard. Significance is set as *P* < 0.05.

**Table 4:** Multiple comparisons between the four groups.

Group (I)	Group (J)	Mean difference (I−J)	*P* value
Healthy control	Myoma	−16.85	0.00
	Benign tumor	−21.13	0.00
	Ovarian cancer	−78.28	0.00

Myoma	Healthy control	16.85	0.00
	Benign tumor	−4.28	0.27
	Ovarian cancer	−61.43	0.00

Benign tumor	Healthy control	21.13	0.00
	Myoma	4.28	0.27
	Ovarian cancer	−57.15	0.00

Ovarian cancer	Healthy control	78.28	0.00
	Myoma	61.43	0.00
	Benign tumor	57.15	0.00

The statistical analysis of these four groups was performed with SPSS for windows 13.0 using one-way AVONA analysis of variance. Significance is set as *P* < 0.05.
